# Systemic EBV+ T-Cell Lymphoma of Childhood with Hemophagocytic Lymphohistiocytosis in a Patient with a Highly Complex Karyotype

**DOI:** 10.3390/genes16040382

**Published:** 2025-03-27

**Authors:** Patrick Maher, Emilia Guzman, Joanna Chaffin, Reema Kashif, Rachel D. Burnside

**Affiliations:** 1 Department of Pathology, Immunology and Laboratory Medicine, University of Florida, Gainesville, FL 32608, USA; patmaher24@ufl.edu (P.M.); jomchaffin@ufl.edu (J.C.); 2 Department of Pediatric Oncology, University of Florida, Gainesville, FL 32608, USA; emiliaguzman@peds.ufl.edu (E.G.); reema.kashif@ufl.edu (R.K.)

**Keywords:** cytogenetics, karyotype, EBV, T-cell lymphoma, HLH, SEBVTCL, chromosome analysis

## Abstract

Background/Objective: Epstein-Barr Virus (EBV) infection can be associated with lymphocytic hematological malignancies, including systemic Epstein-Barr virus-positive T-cell lymphoma of childhood (SEBVTCL). A common complication of EBV infection, hemophagocytic lymphohistiocytosis (HLH), is a life-threatening condition of immune activation present in virtually all cases of SEBVTCL that requires urgent treatment, as this malignancy can be rapidly fatal. Abnormal karyotypes have been strongly associated with SEBVTCL as a distinguishing feature from HLH in the literature. Here, we discuss the diagnostic challenges and social complications in the case of an unaccompanied minor immigrant patient with a highly complex karyotype diagnosed with SEBVTCL with associated HLH. Methods: Laboratory testing confirmed the presence of EBV+ HLH and cytogenetic analysis was performed to investigate a neoplastic process in this patient, confirming SEBVTCL. Chromosomal microarray (CMA) was performed to try to clarify the complex findings by chromosome analysis but demonstrated normal results. Results: Chromosome analysis demonstrated a highly complex hypertriploid clone that confirmed the diagnosis of SEBVTCL. After declining treatment, the patient was discharged to his guardian against medical advice and succumbed to his disease shortly after. Conclusions: SEBVTCL can be challenging to diagnose due to the similarity in clinical and pathological presentations. In virtually all cases reported in the literature, an abnormal karyotype has been reported to be the most important prognostic factor. We propose that in cases with diagnostic ambiguity, an abnormal karyotype can help favor SEBVTCL over EBV+ HLH.

## 1. Introduction

Primary EBV infection often presents as self-limiting infectious mononucleosis (IM) characterized by fever, fatigue, lymphadenopathy, and hepatosplenomegaly, particularly in adolescents and young adults. However, EBV infection can also be associated with the development of secondary malignant lymphoproliferative neoplasias, including B-ALL, classic Hodgkin lymphoma, Burkitt lymphoma, and SEBVTCL of childhood, among others [1,2]. SEBVTCL of childhood is a rare T-cell neoplasm that occurs in the setting of primary Epstein-Barr virus (EBV) infection or chronic active EBV disease, with a median age of 20 years at diagnosis [3]. It disproportionately affects Asian and indigenous populations from Central and South America where EBV is endemic, possibly due to predisposing variants in genes related to immune response within these ethnic/geographic populations [3,4,5]. The typical T-cell phenotype is characterized by clonal CD3(+)/CD8(+)/EBV(+) with aberrant loss of CD5 and CD7 [4]. The high mortality of this diagnosis is due in part to the prevalence of HLH associated with this disease.

HLH is a hyper-inflammatory condition that often results in multi-organ failure and death. It is characterized by a disrupted and exaggerated immune response of NK cells, T cells, and macrophages, usually in response to a genetic mutation or an antigenic stimulus, like a virus or a pre-existing malignancy [6]. HLH is considered a primary diagnosis when it is the result of constitutional pathogenic variants in genes involved in immune response, such as *PRF1*, *UNC13D*, and *STX11* [7], and a secondary diagnosis when it is due to an antigenic stimulus, such as EBV infection—one of the most common causes of secondary HLH. Patients with HLH often present with fever, cytopenia, splenomegaly, and hypertriglyceridemia. The prognosis for children affected with HLH is nearly universally fatal if left untreated. When treated, studies have shown children have a 55% 3-year survival rate and a 22% 5-year survival rate [8]. Distinguishing between SEBVTCL and EBV+ HLH can prove challenging due to similar clinical presentations, along with the tendency of reactive T cells in HLH to show phenotypic aberrancy and, on occasion, clonality by molecular methods [4,9]. Structural chromosomal abnormalities are often seen in SEBVTCL of childhood, but no definitive cytogenetic aberrations have been consistently linked to the condition [10]. However, SEBVTCL of childhood with chromosomal aberrations has been shown to be nearly 100% fatal [10,11,12,13,14,15,16,17]. While it can be challenging to differentiate SEBVTCL of childhood from EBV+ HLH, an abnormal karyotype is most clearly associated with SEBVTCL of childhood and portends an abysmal prognosis.

## 2. Detailed Case Description

A 17-year-old male from an Indigenous Mayan population in Guatemala arrived unaccompanied in the United States approximately 2 months before admission. He was transferred to the emergency room from an outside hospital after presenting with malaise, fever, chest pain, nausea/vomiting, abdominal pain, and jaundice. On the day of admission to our facility, he was in multiorgan failure, presenting with renal failure (Cr = 3.21 mg/dL), bone marrow suppression with pancytopenia (WBC = 1.1 k/µL, plt = 53 k/µL, Hct = 27.4%), and acute liver failure with mild coagulopathy and direct hyperbilirubinemia. He required vasopressors for management of hypotension upon arrival in the ICU.

His presenting symptoms were concerning for primary versus secondary HLH, and he was referred for bone marrow biopsy and pathologic review to evaluate for underlying malignancy. He met multiple HLH-2004 criteria for HLH [18], including fever, pancytopenia, hypertriglyceridemia, hyperferritinemia, hepatosplenomegaly, increased soluble CD25 concentration, and marrow hemophagocytosis. During his hospital course, he received multiple blood products, including blood, platelet, and cryoprecipitate infusions, to treat his pancytopenia. A computed tomography scan was obtained of his chest, abdomen, and pelvis, which showed multiple hyperechoic lymph nodes in the lower neck, axillary, mediastinal, and abdominal areas. The largest lymph nodes were noted in the mediastinum and upper abdomen. While he initially presented with mild ascites and bilateral pleural effusion, he did not require oxygen supplementation throughout his hospital stay. For uremia, he was given multiple doses of 25% 25 g albumin infusions, which led to improvement in his creatinine values. He additionally required frequent electrolyte replacements. The patient was also found to have a positive GI PCR for enteroaggregative *E. coli*, with diarrhea that improved throughout admission. Because HLH can cause organ dysfunction associated with a hyper-inflammatory immune response, an echocardiogram was performed to assess cardiac function, which showed left ventricular dilation and dilated coronary arteries.

Given the clinical diagnosis of HLH, the patient was started on treatment, including anakinra, steroids, intravenous immunoglobulin (IVIG), rituximab, and gamifant. Despite these measures, this patient continued to require a high level of care, though he did show modest clinical improvement. Due to communication barriers, an interpreter fluent in Mayan dialects assisted in conveying the severity of his condition, the high morbidity and mortality of his diagnosis, and the need for urgent intensive treatment. Even with the services of a translator, communication of medical and scientific terminology was challenging. Unfortunately, the patient and his legal guardian did not wish to pursue further treatment in the hospital due to religious reasons. A consultation with Ethics was requested to aid in decision-making; however, because of overall poor health literacy and a cultural/faith-based approach to his diagnosis, the patient and his proxy refused further care. The patient was hospitalized at our institution for a total of 13 days and ultimately was discharged against medical advice.

## 3. Results

Clinical and lab findings were consistent with HLH secondary to systemic EBV (+) T-cell lymphoma of childhood, and he met multiple HLH-2004 criteria for HLH [19]. The results of clinical and laboratory evaluation are summarized in Table 1.

**Figure 1:**
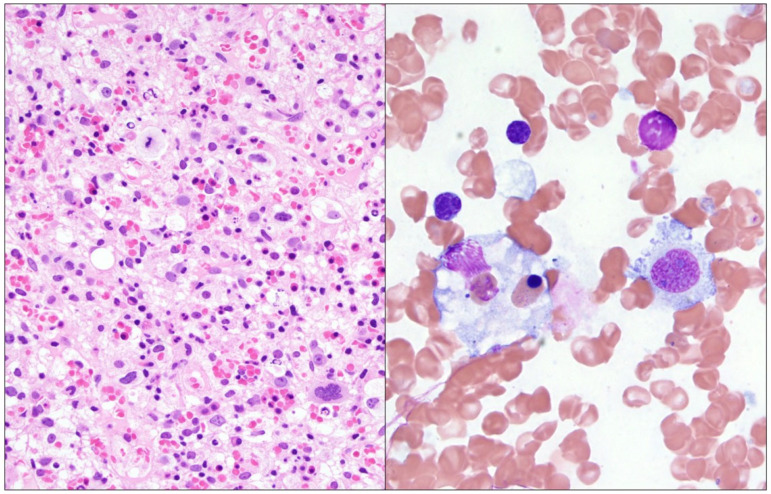
**Left**: Decalcified bone marrow biopsy (hematoxylin and eosin) showing decreased trilineage hematopoiesis and scattered large, atypical cells with prominent nucleoli. **Right**: Bone marrow aspirate (Wright Giemsa) demonstrating a macrophage engulfing a nucleated erythrocyte.

**Figure 2:**
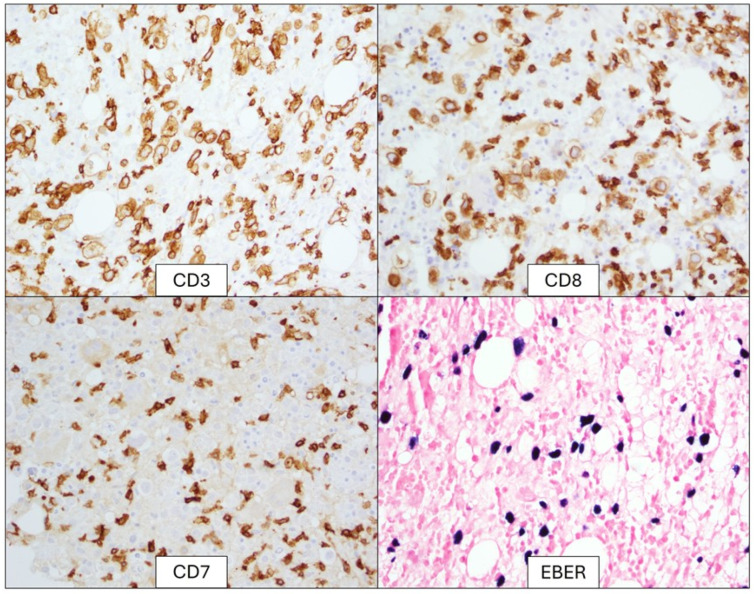
Immunohistochemical stains show large, atypical cells expressing CD3 and CD8 while lacking CD7 expression. The large, atypical cells are uniformly positive on EBER in situ hybridization.

**Figure 3:**
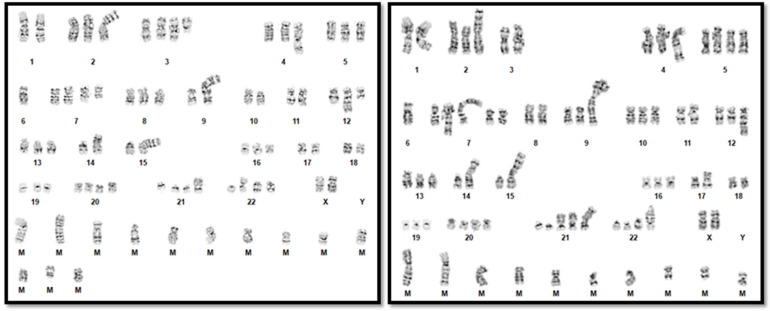
Representative karyograms demonstrating karyotype complexity.

EBV PCR performed on peripheral blood was positive. The bone marrow biopsy demonstrated an atypical infiltrate of large, atypical lymphocytes with prominent nucleoli in addition to hemophagocytic cells (Figure 1). Immunohistochemical and in situ hybridization staining showed positive expression of CD3, CD8, and Epstein-Barr Encoding Region (EBER) but lacked CD7 expression (Figure 2). Flow cytometry of the bone marrow aspirate confirmed the presence of an aberrant T-cell population positive for CD3 and CD8 but negative for CD4, CD5, CD7, CD56, and CD57. T-cell receptor-gamma PCR detected a clonally rearranged population. Chromosome analysis demonstrated a highly complex hypertriploid clone: 71~79,XX,-Y,-1,add(1)(q12),add(2)(p13),-3,add(4)(q35),+5,-6,dic(6;15) (q27;p13),+7,+7,add(7)(p22), del(7)(q31),der(7)t(2;7)(p11.2;q11.2),add(8)(p22),add(9)(q34),der(9;9)(9qter→9p22::?::9p22→9qter::?),add(11)(q13),der(11)t(9;11)(q11.2;q13),add(12)(q21),-14,der(14;17)

(17qter→17p13::?::14p13→14qter),-15,-15,add(15)(p12),add(16)(q13),add(17)(p11.2), der(17)(3qter->3q13.1::?::17p13->17qter),−18,+20,+20,+21,+21,+21,add(21)(p11.2), dic(21:?)(p13;?)x2,add(22)(p12),+1~6mar[cp12]/46,XY[3] by ISCN 2020 [18] (Figure 3). To try to clarify the cytogenetic results, subsequent CMA analysis on DNA extracted from fixed cell pellets was performed, with normal results. It is possible that the abnormal cells were preferentially analyzed in the chromosome analysis but that the percentage of abnormal cells was below the limit of detection for CMA.

Taken together, the results were most consistent with a diagnosis of SEBVTCL of childhood, supported by the abnormal karyotype. After discharge against medical advice, the patient succumbed to his disease approximately one week later.

## 4. Discussion

This case was diagnostically challenging, because unlike B-cell lymphomas, in which kappa and lambda restriction offers easy, strong support for clonality, clonality in T cells is more difficult to identify, is non-specific to SEBVTCL or EBV+HLH, and can often demonstrate false-positive results in a reactive setting. Phenotypic T-cell aberrancy, like loss of CD45, dim expression of pan T-cell antigens, and CD4/CD8 dual expression or dual loss, can be helpful, but these features may also be seen in reactive T-cell expansion and are not definitive [20,21]. In a 2014 review by Smith et al., the authors analyzed outcome data for 74 EBV+ HLH cases and 20 systemic EBV+ lymphoproliferative disorders and discovered evidence of monoclonality in both entities in 60 patients [11]. The authors suggested that the presence of an abnormal karyotype may carry more significant prognostic information than other assessments of clonality and proposed that an abnormal karyotype may be used to distinguish SEBVTCL from EBV-HLH. Moreover, patients with abnormal karyotypes were universally fatal, regardless of diagnosis [11,12]. Since the Smith publication in 2014, additional reports of patients with abnormal karyotypes have been published [13,14,15,16,17,22]. Given the lack of recurrent chromosomal abnormalities associated with SEBVTCL, it is possible abnormal karyotypes reflect general neoplasia-related genomic instability, thus portending a worse prognosis. A single report in the literature describes an adolescent white male diagnosed with EBV-HLH with an abnormal karyotype who survived treatment and remained in remission after 3 years [15]. We are not aware of any reports of survivors of SEBVTCL with an abnormal karyotype.

## 5. Conclusions

**Figure 4:**
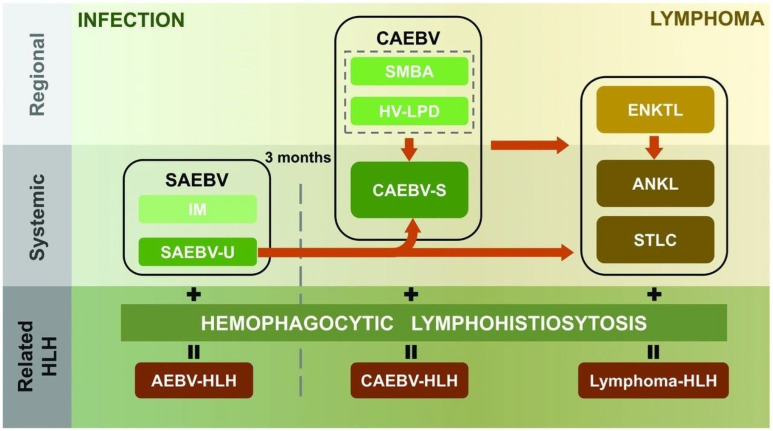
Proposed spectrum of EBV-associated T/NK lymphoproliferative disorders by Chen and Guan, from self-limiting infections to lymphoma. HLH may be present at any point along the continuum. SAEBV—systemic active EBV infection; IM—infectious mononucleosis; SAEBV-U—systemic acute EBV infection-unclassifiable; AEBV-HLH—active EBV infection-HLH; CAEBV—chronic active EBV infection; SMBA—severe mosquito bite allergy; HV-LPD—hydroa vacciniforme-like lymphoproliferative disorders; CAEBV-S—chronic active EBV infection-systemic form; ENKTL —extranodal NK/T-cell lymphoma; ANKL—aggressive NK-cell leukemia; STLC—systemic EBV-positive T-cell lymphoma of childhood. Used with Permission by Ferrata Storti Foundation. All rights are reserved to the ©2025 Ferrata Storti Foundation.

SEBVTCL of childhood is a diagnostic entity that was introduced in the revised 4th edition of the WHO published in 2016. The similarities between SEBVTCL of childhood and EBV+ HLH have been extensively reported, and an evolving definition of the entity has made it difficult to interpret the literature, where systemic EBV(+) T-cell lymphoproliferative disorder also included many obsolete diagnoses, such as fulminant EBV+ LPD of childhood, sporadic fatal infectious mononucleosis, fulminant hemophagocytic syndrome in children, and severe chronic active EBV infection. Fernandez-Pol et al. (2018) proposed a continuum of diagnoses from infections to neoplasia, and in the same issue, Chen and Guan proposed a modification to include acute systemic non-neoplastic infections without HLH and acute systemic pre-malignant infections (Figure 4) [23,24]. Moreover, clonal cytogenetic abnormalities seem to favor SEBVTCL of childhood, consistent with the proposal by Smith et al. [11].

The patient in this report followed the nearly universal fatal outcome of patients diagnosed with SEBVTCL of childhood with an abnormal karyotype. Regardless of diagnosis and consistent with current diagnostic algorithms, the presence or absence of a cytogenetic abnormality appears to be the most important prognostic factor in both EBV+ HLH and SEBVTCL of childhood. In cases where diagnostic ambiguity remains, abnormal cytogenetic results may help to discern a diagnosis of SEBVTCL. While clonality has been observed in EBV-HLH, cytogenetic aberrations are more specific for SEBVTCL [4].

## Figures and Tables

**Table 1:** Clinical and laboratory findings. Bolded findings are positive criteria for HLH per HLH-2004.

Parameter	Finding/Value	Reference Range
Temperature	**39.4 °C**	36.1–37.2 °C
Hemoglobin	**8.0 g/dL**	12.0–16.0 g/dL
Platelet count	**53 × 10^3^ µL**	150–400 × 10^3^ µL
Absolute neutrophil count	**0.72 × 10^3^ µL**	1.7–7.00 × 10^3^ µL
Triglycerides	**608 mg/dL**	<150 mg/dL
Fibrinogen	**166 mg/dL**	173–454 mg/dL
Ferritin	**>13,000 ng/mL**	11–204 ng/mL
Splenomegaly	**22.7 cm**	<12 cm
Soluble CD25	**66,395.7 pg/mL**	175.3–858.2 pg/mL
CXCL9	89,046 pg/mL	<647 pg/mL
EBV Quant PCR	218,658 IU/mL	Not detectable
Hemophagocytosis in marrow	**Present**	Absent

Note: The patient met 6 out of 8 criteria for hemophagocytic lymphohistiocytosis based on HLH-2004 guidelines, including fever, pancytopenia, hypertriglyceridemia, hyperferritinemia, splenomegaly, increased soluble CD25 concentration and marrow hemophagocytosis.

## Data Availability

All relevant data is in the case report.
